# Which predictive variables are emphasized when violence risk assessments are performed in Norway?

**DOI:** 10.1192/j.eurpsy.2022.1542

**Published:** 2022-09-01

**Authors:** K. Seljenes, R. Wynn

**Affiliations:** UiT The Arctic University of Norway, Department Of Clinical Medicine, Tromsø, Norway

**Keywords:** Violence risk assessment, Primary health care, Norway, Emergency psychiatry

## Abstract

**Introduction:**

Violence is considered both a societal issue and a public health issue. Due to the high economic, societal, and individual cost associated with exposure to violence, clinical risk assessment-tools are now being implemented in the public health care system as well as outside of it. To ensure early identification and prevention, various professional groups perform structured risk assessments in Norway, including police, doctors, and psychologists.

**Objectives:**

There is a need to examine competence and organizational factors, which may affect the ability to make accurate assessments in different levels of the health service, as well as in the police who often are involved in early identification and action-taking concerning violent individuals. Based on variation in risk assessment competencies, and characteristics of different work environments, our project aims to investigate whether some factors seem to be more important than others in clinical assessments when comparing different professional groups with or without a professional background in health care.

**Methods:**

In our study, we will be able to tell if there is a significant difference in how different professional groups emphasize different risk factors, and in which way individual factors such as formal competencies, years of experience, and personal and professional attitudes to violence affect the risk violence assessments performed.

**Results:**

We hypothesize that retrospective, clinical, and dynamic risk-factors are interpreted differently by different professional groups, and therefore entail significant variations in assessments, and the health care provided.

**Conclusions:**

In this planned study, we will examine variations in the practice of violence risk assessment in Norway.

**Disclosure:**

No significant relationships.

